# Expression and Immunogenic Application of Recombinant DENV-2 rprM Protein for the Non-Invasive Detection of Salivary IgA Antibodies

**DOI:** 10.3390/ijms27188087

**Published:** 2026-09-11

**Authors:** Julio García-Cordero, Luis Alberto Sánchez-Vargas, Rosendo Luria-Pérez, Héctor Vivanco-Cid, Leticia Cedillo-Barrón, Jazmín García-Machorro, Gabriela Mellado-Sánchez

**Affiliations:** 1 Departamento de Biomedicina Molecular, Centro de Investigación y Estudios Avanzados (CINVESTAV-IPN), Mexico City 07360, Mexico; 2 Centro de Investigaciones Científicas, Instituto de Biotecnología, Universidad del Papaloapan, Oaxaca 68301, Mexico; lsanchez@unpa.edu.mx; 3 Department of Cell and Molecular Biology, Institute for Immunology and Informatics, University of Rhode Island, Providence, RI 02903, USA; 4 Unidad de Investigación en Enfermedades Oncológicas, Hospital Infantil de México Federico Gómez, Mexico City 06720, Mexico; rluria@himfg.edu.mx; 5 Instituto de Investigaciones Médico-Biológicas, Universidad Veracruzana, Veracruz 91700, Mexico; hvivanco@uv.mx; 6 Laboratorio de Medicina de Conservación, Escuela Superior de Medicina, Instituto Politécnico Nacional, Mexico City 11340, Mexicojgarciama@ipn.mx (J.G.-M.); 7 Unidad de Desarrollo e Investigación en Bioterapéuticos (UDIBI), Escuela Nacional de Ciencias Biológicas, Instituto Politécnico Nacional, Mexico City 11340, Mexico; 8 Laboratorio Nacional para Servicios Especializados de Investigación, Desarrollo e Innovación (I+D+i) para Farmoquímicos y Biotecnológicos, LANSEIDI-FarBiotec-CONACyT, Prolongación de Carpio y Plan de Ayala S/N, Colonia Santo Tomás, Alcaldía Miguel Hidalgo, Mexico City 11340, Mexico

**Keywords:** viral infection, dengue, prM protein, antiviral immunity, antigenicity, antibody response, salivary IgA, non-invasive approach, proof-of-principle, innovation

## Abstract

Dengue virus (DENV) serotypes 1–4 cause dengue, a major re-emerging disease in tropical and subtropical regions. This study evaluated the antigenicity of a recombinant DENV-2 premembrane protein (rprM-DENV-2) and its ability to detect anti-DENV human IgA antibodies. The rprM-DENV-2 protein was expressed in *Escherichia coli*, purified, and assessed for immunogenicity and antigenicity. The recombinant protein induced IgG antibodies in rats, which detected DENV infection in Vero cells. Importantly, in paired saliva and serum samples from patients diagnosed with dengue, IgA antibodies were detected by ELISA using the rprM-DENV-2 antigen, but not in samples from the negative control group (subjects residing in a non-endemic area), with a significant difference (*p* < 0.0001). These findings demonstrate that rprM-DENV-2 retains antigenic properties similar to the native viral protein; this may be useful for in vitro studies of DENV infection and IgA detection in non-invasive clinical samples.

## 1. Introduction

Dengue virus (DENV) infection remains the most significant mosquito-borne viral disease worldwide, with an increasing global burden and a complex clinical spectrum [1]. The etiological agents are the four serologically and genetically related types of DENV, known as DENV1-4, which belong to the Flaviviridae family, *Orthoflavivirus* genus [2,3]. In 2009, the World Health Organization (WHO) redefined the classification of the disease into dengue without warning signs (No WS), dengue with warning signs (WS), and severe dengue to improve clinical management and early identification of patients at risk of complications [4]. Accurate and timely diagnosis is crucial, particularly during the transition from the febrile to the critical phase, where warning signs manifest, and the risk of plasma leakage and hemorrhage increases [5].

The DENV particle consists of a single-stranded positive-sense RNA surrounded by an icosahedral capsid and an envelope. It encodes three structural proteins, namely capsid (C), premembrane (prM), and envelope (E), as well as seven non-structural proteins [6]. While the E protein is the main mediator of receptor binding and the primary target of neutralizing antibodies, the prM protein has garnered attention due to its association with pathology [7]. During DENV infection, incomplete cleavage of prM during virion maturation leads to the release of a heterogeneous population of mature, partially mature, and immature viral particles that retain prM on their surface, resulting in extensive exposure of prM epitopes to the host immune system [8].

Because of this structural and maturation heterogeneity, prM elicits a robust and highly prevalent antibody response in naturally infected individuals. Anti-prM antibodies are broadly cross-reactive among DENV serotypes and constitute a substantial fraction of the total anti-DENV humoral response, making prM a reliable target for serological detection despite its limited neutralizing capacity [9]. Importantly, recent studies have demonstrated that prM-based antigens can be successfully incorporated into serological assays for dengue diagnosis [10], supporting the utility of prM as a complementary antigen to classical E- and NS1-based diagnostic approaches [11]. Given the abundance and consistency of prM-directed antibody responses during natural infection, prM is a strong candidate antigen for detecting alternative antibody isotypes, including IgA, in both invasive and non-invasive clinical specimens.

Conventional laboratory diagnosis of DENV infection is based on viral isolation, detection of the viral genome (molecular tests during the first 0–7 days of the course of the disease) or viral antigens (such as non-structural protein 1 (NS1)), and serological detection of dengue-specific antibodies (IgM antibodies) [9], which identifies recent infections after day 3 of illness. Antibodies from past dengue or other *Orthoflavivirus* infections, such as IgG antibodies, can also be detected; however, they are not recommended for diagnosing acute dengue in patients, since seroconversion during a first infection occurs approximately 14 days after onset. An increase in IgG levels of more than fourfold between two samples can also indicate a recent infection [12].

On the other hand, non-invasive clinical specimens such as saliva and urine have increasingly been explored as alternatives to blood collection [13]. DENV-specific IgA antibodies can be detected not only in serum of infected individuals, but also in saliva and urine. For example, IgA kinetics in serum and saliva have been described in acute infections [13,14]. Furthermore, detection of DENV-specific IgA in urine has been associated with disease severity, with higher positivity rates and signal intensity observed in severe dengue compared with non-severe cases, suggesting a potential role for IgA not only in diagnosis but also in prognostic stratification [15]. A recent meta-analysis indicates that IgA-based tests demonstrate moderate diagnostic accuracy, with performance influenced by timing of sample collection and infection status [16].

This proof-of-principle study aims to evaluate the recombinant prM protein from DENV-2 (prM-DENV-2) obtained in a bacterial expression system, and explore its potential for two applications: (1) use as an antigen to produce a polyclonal antibody that can be used in the detection of DENV infection in cell cultures (indirect antigenicity); and (2) potential use as an antigen in an ELISA to detect specific IgA-class antibodies in patient specimens. The second part of this study focuses on an unexplored area of immunity induced during DENV infection: the IgA responses elicited by the prM protein.

## 2. Results

### 2.1. Construction of prM-DENV-2 Recombinant Plasmid

**Figure 1:**
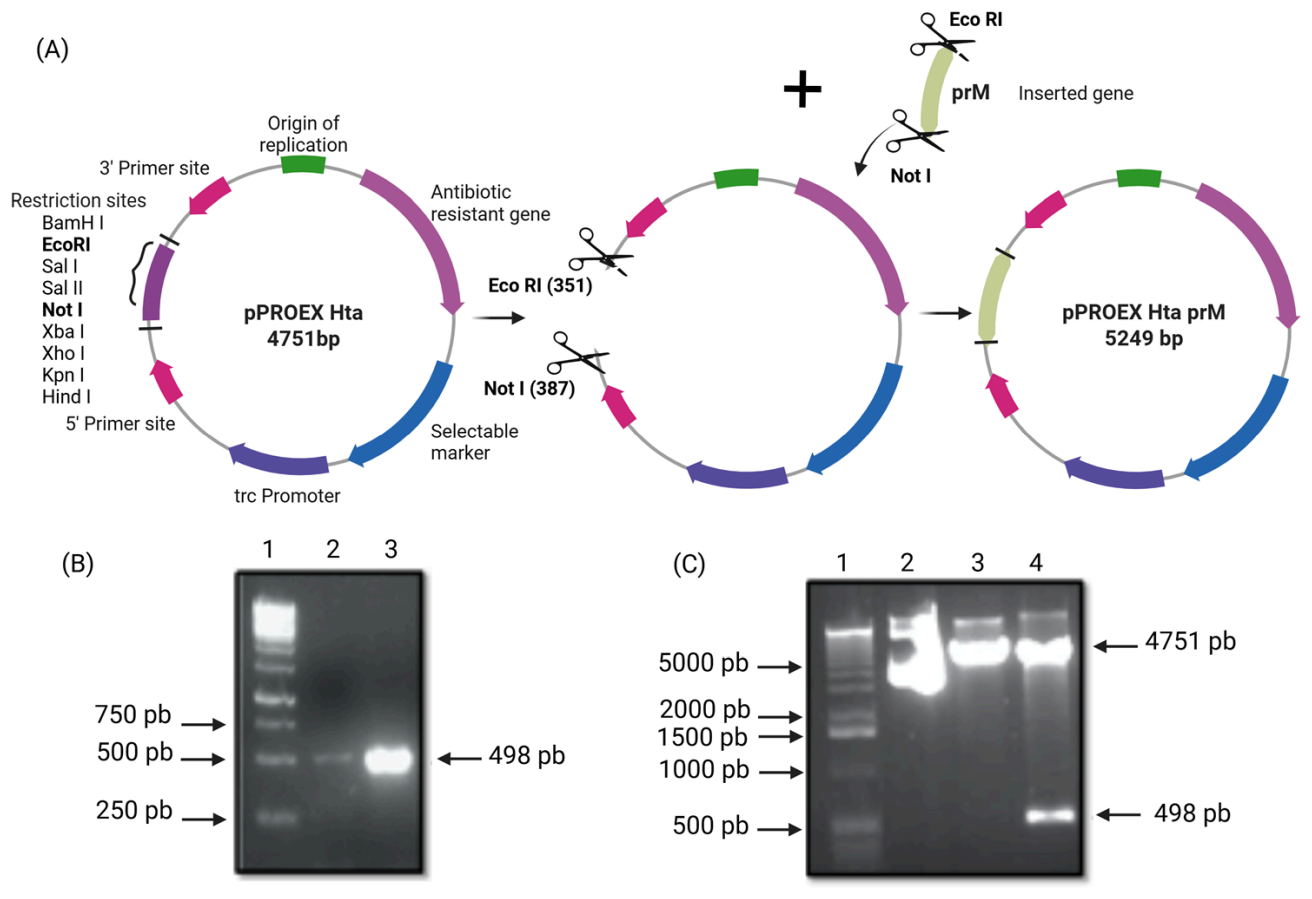
prM gene of DENV-2 was cloned into a prokaryotic expression vector. (**A**) Diagram of the plasmid pPROEX HTa and depiction of digestion with the restriction enzymes EcoRI and NotI. Created in BioRender. (**B**) The PCR product of rprM-DENV-2 was analyzed on a 1.2% (*w*/*v*) agarose gel: lane 1, molecular weight marker (MWM); lane 2, 1 µL of the template; lane 3, 2 µL of the template. (**C**) Restriction analysis of the plasmid pPROEX HTaprM on a 1% (*w*/*v*) agarose gel: lane 1, MWM; lane 2, undigested plasmid pPROEX HTaprM; lane 3, digested pPROEX HTaprM plasmid with EcoRI; lane 4, pPROEX HTaprM plasmid digested with the enzymes that flanked the insert (EcoRI and NotI) releasing a fragment of 498 bp.

The gene encoding the recombinant prM-DENV-2 (rprM-DENV-2) protein includes prM residues from amino acids 6 to 166, derived from GenBank accession CAA51363.1. This sequence was PCR-amplified from cDNA of C6/36 cells infected with DENV-2. The fragment with the projected size of 498 bp (Figure 1B) was cloned into the pPROEX HTa vector (Figure 1C). Restriction analysis was performed on the novel construct to confirm the presence of a prM-DENV-2 insert in the plasmid pPROEX HTa (Figure 1C). DNA sequencing was employed to identify and to verify the orientation of the fragment. The cloning strategy is depicted in Figure 1A.

### 2.2. Expression and Identification of rprM-DENV-2 Protein

**Figure 2:**
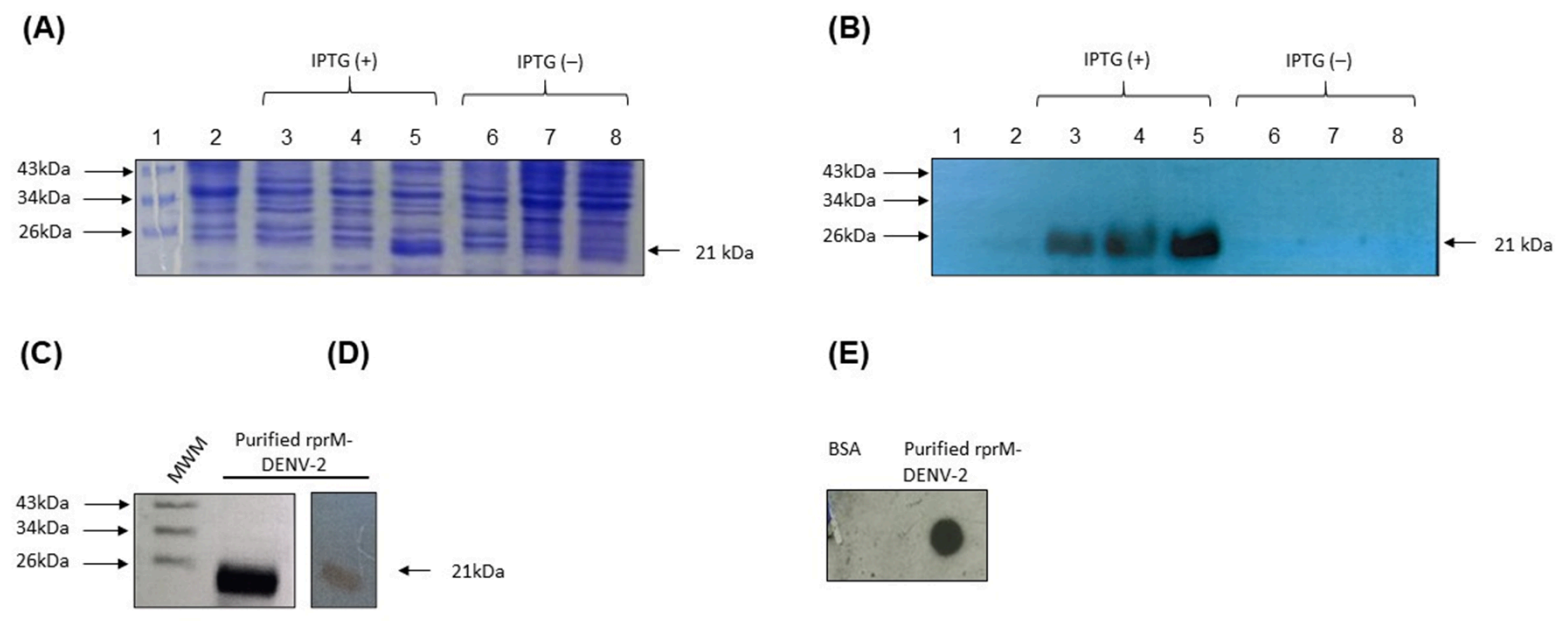
The rprM-DENV-2 protein was detected in bacterial lysates, and identified in purified protein. The recombinant protein was assessed using (**A**) SDS-PAGE with Coomassie staining and (**B**) Western blot to establish the expression kinetics after the addition of IPTG. Culture aliquots were collected at different times, and the bacteria were lysed. Lane 1, molecular weight marker; lane 2, culture just before adding IPTG; lanes 3, 4, and 5, culture induced at 3 h, 6 h, and 12 h post-induction, respectively; lanes 6, 7, and 8, non-induced culture at the previously mentioned times. After purification with Ni-NTA beads, the purified rprM-DENV-2 protein was subjected to the following techniques. Firstly, eluted proteins were separated via (**C**) SDS-PAGE, and the resulting gel was stained with Coomassie to visualize the protein bands. Alternatively, (**D**) a Western blot was performed, employing a specific anti-His antibody to detect the target protein, followed by revealing the antibody (anti-IgG-HRP) and using diaminobenzidine as the chromogenic substrate. In addition, (**E**) a dot blot assay was carried out using bovine serum albumin (BSA) as a negative control, along with purified rprM-DENV-2, on a PVDF membrane. The membrane was then probed with an anti-prM antibody and detected with an anti-mouse IgG1-HRP antibody. After the recombinant protein was characterized and its identity confirmed, the production was increased, and purification was performed using Ni columns. The average yield of the recombinant protein produced in this system was 8 mg/mL for rprM-DENV-2.

To evaluate the expression of rprM-DENV-2 given by the *trc* promoter (a hybrid of the *lac* and *trp* promoters), the protein was labeled with a 6×His tag at the N-terminus. The lysates, obtained at different times post-induction from the transformed bacteria, were evaluated on SDS-PAGE gels. The rprM-DENV-2 was evinced after Coomassie blue staining (Figure 2A). The kinetics were also evaluated by Western blot using an anti-His antibody. Here, the protein was detected in lysates obtained after the first 3 h; it corresponds to a 21 kDa band, consistent with the predicted size of rprM-DENV-2 (Figure 2B). The rprM-DENV-2 was purified using nickel NTA beads, and SDS-PAGE, followed by Coomassie staining, was used to determine the protein purity. The final purified antigen was highly pure for rprM-DENV-2 (Figure 2C). A Western blot confirmed that the 21 kDa band belongs to a protein with a His tag (Figure 2D). Additionally, the dot blot analysis demonstrated that the purified protein was indeed prM-DENV-2 and retained a conformational epitope similar to the native one, which was recognized by the monoclonal antibody anti-prM-DENV (Figure 2E).

### 2.3. Indirect Evaluation of Antigenic Properties of rprM-DENV-2

#### 2.3.1. Generation of Anti-rprM-DENV-2 Hyperimmune Rat Serum

**Figure 3:**
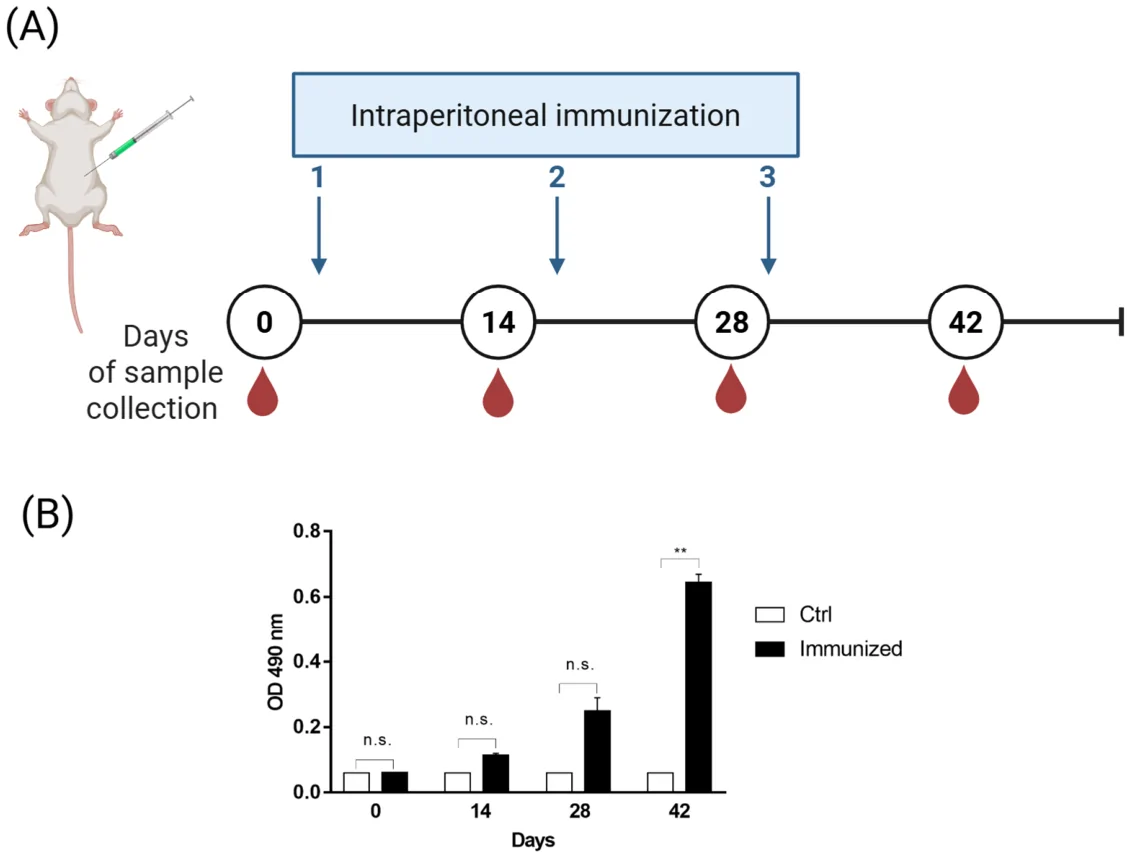
rprM-DENV-2 stimulates specific antibody responses in rats. (**A**) Immunization protocol. Three rats were immunized with 100 μg of rprM-DENV-2 on days 0, 14, and 28. Serum samples were collected and individually tested on days 0, 14, 28, and 42 post-first immunization. Created in BioRender. (**B**) Kinetics of the induction of anti-rprM antibodies in rats. The sera from the immunization protocol were analyzed by indirect ELISA, in which rprM-DENV-2 was used as the antigen and horseradish peroxidase-conjugated goat anti-rat IgG as the secondary antibody. The respective preimmune serum samples were also analyzed. All sera were diluted 1:400. Data are expressed as mean ± SEM. Multiple *t*-tests were carried out to compare each time and group, ** *p* < 0.01 and n.s. no significant difference.

In this analysis, rprM-DENV-2 was inoculated in three Wistar rats according to the immunization protocol depicted in Figure 3A. All serum samples were analyzed by ELISA. As observed in Figure 3B, a specific anti-prM-DENV-2 IgG response was detected at 1:400 serum dilution (the remaining dilutions yielded higher ODs)). Hyperimmune serum samples showed a more robust response than preimmune serum samples, whilst control antisera (PBS) evinced a minimal response. These data indicate that rprM-DENV-2 efficiently induced an antigen-specific antibody response.

#### 2.3.2. Evaluation of Anti-rprM-DENV-2 Serum for In Vitro Detection of DENV Infection

**Figure 4:**
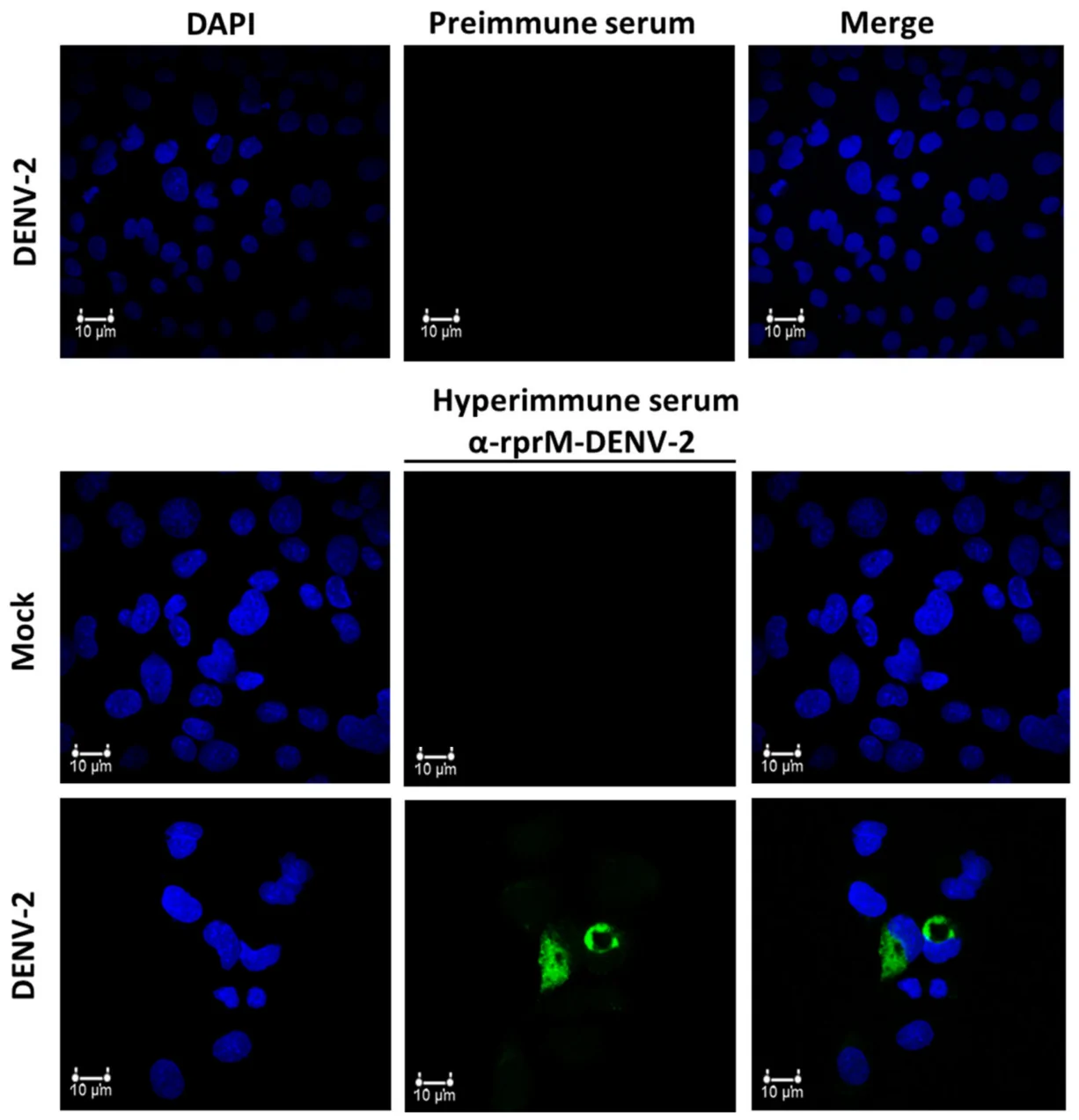
Antibodies from rat hyperimmune serum against rprM-DENV-2 recognize the native prM protein expressed during viral infection. A pool of animal sera from day 42 after the first immunization with rprM-DENV-2 protein, obtained as described in Figure 3, was used for detection of the native antigen in an immunostaining assay. Vero cells were infected with DENV-2 at an MOI of 2, and 24 h after infection, the cells were tested by immunofluorescence, with a 1:100 dilution of rat hyperimmune serum used as the primary antibody and then anti-rat IgGs-Alexa Fluor 488 (green signal) used as the secondary antibody, and nuclei stained with DAPI (blue signal). The figure shows a representative experiment with 40× magnification.

To determine whether antibodies induced in rats immunized with rprM-DENV-2 were capable of detecting native DENV-2, Vero cells were infected at a multiplicity of infection (MOI) of two with DENV-2. Brefeldin A was added to the cells to stop vesicular traffic, and 4 h later the cells were fixed. The cells were then incubated with sera from rats immunized with rprM-DENV-2 at a 1:100 dilution. Evident and specific recognition of DENV by the hyperimmune serum sample was demonstrated by intracellular immunostaining (Figure 4). On the other hand, no positive signal was observed with the preimmune serum samples. This was an indirect way to test that the antigenic properties of rprM-DENV-2 resemble those of the native viral protein; otherwise, the antibodies induced by the recombinant protein could not detect the native protein.

### 2.4. rprM-DENV-2 Protein Antigenicity and Its Usefulness as an Antigen in an ELISA

To assess the properties of rprM that are recognized by anti-DENV IgA antibodies (antigenicity), samples from the serum and saliva of dengue patients were analyzed using an indirect ELISA. It is essential to highlight that all patients experienced a secondary DENV infection (See File S1. Raw data file); thus, the priming serotype was unknown, but the cause of the secondary infection could be assumed since, during the study period (June–August 2014), all serotypes of DENV were circulating in Veracruz state, a tropical place in Mexico, with serotype 2 being the most frequent.

**Figure 5:**
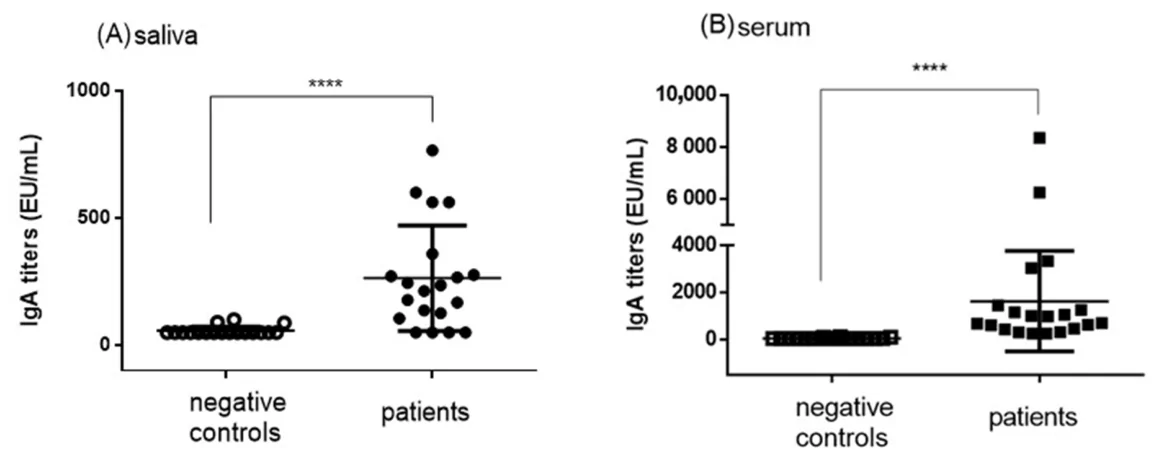
IgA antibodies against the rprM-DENV-2 protein in samples from dengue patients and negative controls. Paired biological samples (saliva and serum) were obtained from 18 negative controls (Mexican non-endemic dengue area) and from 20 dengue patients during an outbreak on the East Mexican Coast (Veracruz) that occurred between Jun and Aug 2014. An indirect ELISA was performed using prM-DENV-2 protein as the antigen, biological samples as the primary antibody, an HRP-labeled goat anti-human IgA as the secondary antibody, and detection with tetramethylbenzidine, with the optical density measured at 450 nm. The *x*-axis shows the negative control group and patients, and the *y*-axis shows IgA titers in ELISA units per mL (EU/mL). (**A**) Circles correspond to saliva samples; (**B**) squares represent serum samples. The means and error bars are depicted. Statistical significance: **** *p* < 0.0001.

The Shapiro–Wilk normality test was used to assess the normality of the IgA antibody detection results in saliva and serum, revealing that the data did not follow a normal distribution (*p* < 0.0001). Therefore, the Mann–Whitney U test was applied to compare the results between the negative control group (samples from subjects residing in Mexico City) and the experimental group (samples from patients previously diagnosed with dengue through commercial immunoassays). The results are expressed as ELISA units per mL (EU/mL), and a significant difference (*p* < 0.0001) was found when comparing IgA antibody titers against the rprM-DENV-2 protein detected in samples from patients diagnosed with dengue versus the control group, both in saliva (Figure 5A) and serum (Figure 5B).

Subsequently, it was determined whether the rprM-DENV-2 antigen could be differentially detected by IgA antibodies in saliva and serum from paired patient samples. Therefore, a matched Wilcoxon signed-rank test was applied. A significant difference (**** *p* < 0.0001) was found between the antibodies detected in saliva (median 225 EU/mL, mean 264 EU/mL) and those detected in serum (median 853 EU/mL, mean 1635.55 EU/mL).

**Figure 6:**
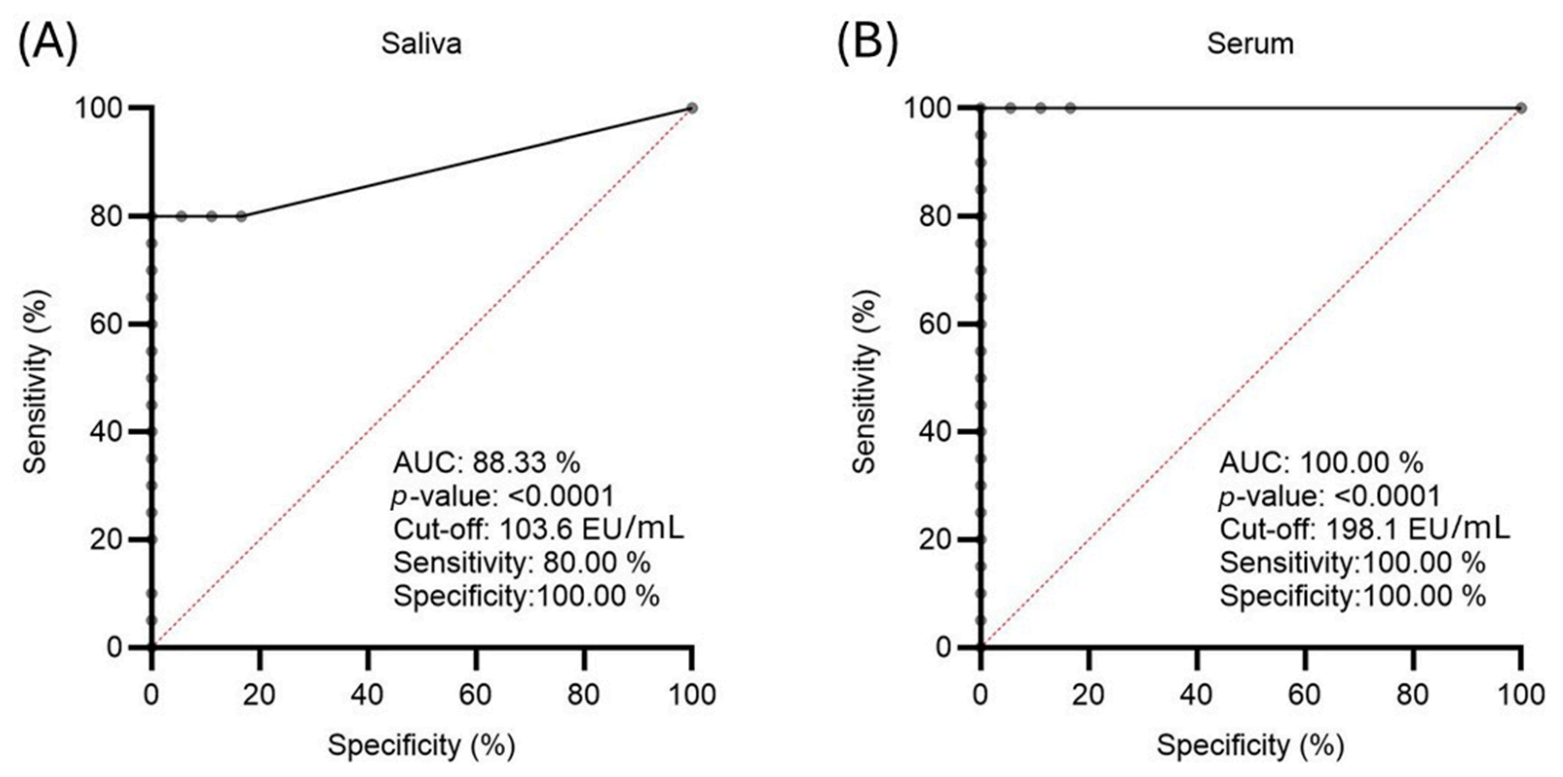
AUROC analysis of anti-rprM-DENV-2-IgA as a non-invasive detection tool for dengue. (**A**) Saliva and (**B**) serum. The area under the Receiver Operating Characteristic curve (AUROC) was determined using a 95% confidence interval. An AUROC > 0.7 was considered acceptable. The red dotted diagonal line represents a test that does not discriminate at all between those with and without disease

To evaluate whether the rprM-DENV-2 antigen could discriminate between healthy control samples and dengue patient samples, sensitivity and specificity were calculated using a Receiver Operating Characteristic (ROC) curve, and they are depicted in Figure 6. This allowed us to identify the cut-off point that provides the best balance between sensitivity and specificity, calculated using the Youden index. The results in saliva samples show a good discriminative ability of the EU/mL to differentiate between individuals with dengue and healthy controls, with an area under the ROC curve (AUROC) of 88.3% and a cut-off point of 103.6 EU/mL, and with a sensitivity of 80% and a specificity of 100%. Furthermore, using this data, the Positive Predictive Value (PPV) and the Negative Predictive Value (NPV) were calculated; they were 100% and 81.8%, respectively (see Table 1). Meanwhile, for serum samples, the AUROC was 100%, and sensitivity and specificity were 100% and 100%, respectively, with a cut-off of 198.1%. PPV and NPV had the same value, 100% (see Table 2).

**Figure 7:**
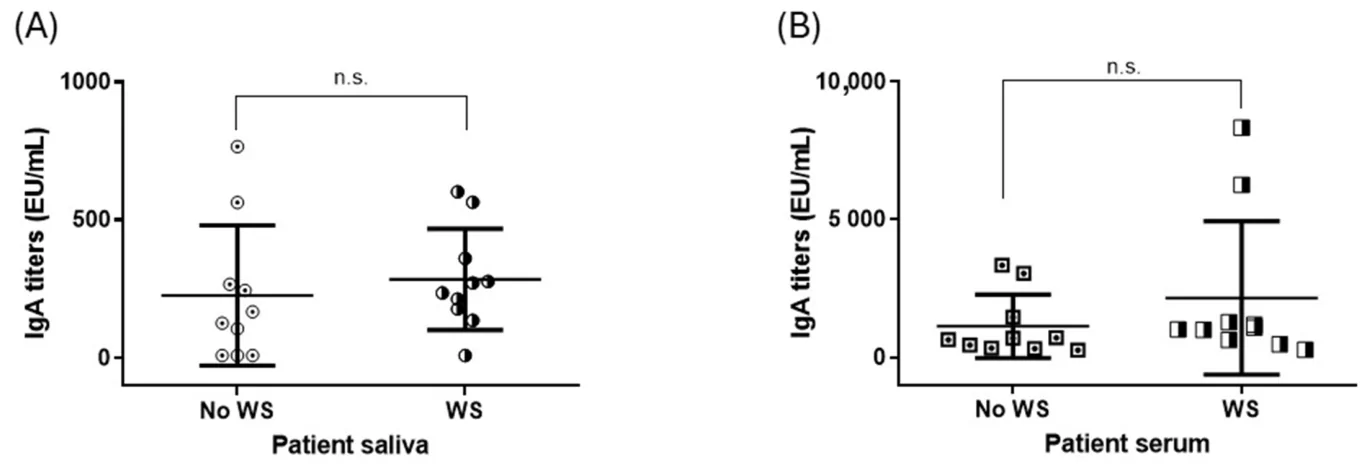
IgA antibodies against the rprM-DENV-2 protein in samples from patients diagnosed with dengue without warning signs (No WS) and with warning signs (WS). The patient data plotted in Figure 5 were reanalyzed. They were then classified by the severity of their clinical manifestations, resulting in two subgroups: 10 patients with dengue No WS and 10 with dengue WS. The *x*-axis shows the negative control group and patients, and the *y*-axis shows IgA titers in ELISA units per mL (EU/mL). (**A**) Circles correspond to patient saliva; (**B**) squares represent patient serum. The mean and error bars are depicted. The scale is linear, and no significant (n.s) difference was found between the groups.

Once the rprM-DENV-2 protein showed differential antigenicity when it was tested with saliva and serum antibodies from patients diagnosed with dengue, the results were analyzed by classifying the samples into two subgroups: those from patients diagnosed with dengue No WS and those diagnosed with dengue WS. The analysis is depicted in Figure 7. No significant difference was found between No WS and WS patients in either the saliva or serum samples.

## 3. Discussion

Dengue is a disease that has been progressively increasing worldwide over the last two decades. It is caused by four genetically related serotypes (DENV 1-4) [1] and encompasses a broad spectrum of clinical manifestations: dengue No WS, dengue WS, and severe dengue [4,17].

Despite many years of research, the pathogenesis of DENV infection remains complex and requires further study. Cases of severe dengue are explained by ADE (antibody-dependent enhancement) [18], which occurs in vivo in secondary infections and may be associated with several risk factors, such as (a) a secondary infection with a different serotype, (b) certain combinations of serotypes during primary and secondary infections, (c) the association of specific genotypes, (d) the interval between the first and second infections, (e) host age, (f) host ethnicity, and (g) chronic disease comorbidities [18]. Therefore, an appropriate characterization of the immune response induced by DENV infection is critical. In this context, using viral recombinant proteins obtained with recombinant DNA technology has fostered the development of in vitro and in vivo models used to analyze the immune response triggered by DENV infection or to design less invasive diagnostic tools [19].

Once DENV enters the body, it induces a broad sero-specific and prolonged humoral immune response, characterized by the presence of specific IgM, IgG, or IgA antibodies against viral structural antigens, particularly protein E [20]; the intensity of the response varies depending on whether the infection is primary or secondary [19]. Several studies have also documented the induction of a predominantly antibody-mediated response against the prM protein [8], a protein that plays an essential role in the maturation of viral particles in encapsidation and packaging [3]; its presence stabilizes glycoprotein E [21] and promotes an immune response against this protein [22]. Studies that have evaluated the use of the prM protein as a tool to characterize the immune response induced by DENV or for diagnostic purposes [9] have revealed efficient detection of IgM and IgG antibodies against the prM protein in the serum of patients who are DENV-positive [23].

Since the presence of specific antibodies for the prM protein predominates in primary and secondary DENV infections in humans [8], and since these are significantly enhanced in patients with a secondary infection in comparison with a primary infection [24], the prM protein could be a potential target for diagnostic testing and for monitoring the induction of the immune response against DENV. Accordingly, with these observations, this study describes an efficient method to produce a DENV-2 prM recombinant protein in an easily manipulated bacterial expression system that yields protein concentrations with an appropriate rearrangement for immunological analysis; this premise is based on the scant glycosylation that is necessary for the protein prM’s structure and function (three possible detected sites but only one with an appropriate glycation score, Supplementary Figure S1).

Several studies have described the need to use complex eukaryotic systems to obtain recombinant proteins that require post-translational modifications, particularly glycosylation, for proper structure and correct function; this is a condition that, in many cases, favors the induction of immune response against these glycoprotein structures, in such a way that the use of the prokaryotic protein production system proposed in this work (with no glycosylation of the proteins) will allow for the rprM protein to be obtained with adequate structure and function, since it does not require extensive glycosylation processes (only three possible glycosylation sites were detected), which will induce immunogenicity and antigenic properties similar to the native protein [25]. Additionally, computational studies have mapped B-cell-specific epitopes of the prM protein and found that glycosylation is not necessary [26]. This supports the results from this study, in which, despite the lack of glycosylation of the rprM-DENV-2 protein, antibody recognition shows a differential response between the serum and saliva of infected patients.

The sequence encoding the prM protein (DENV-2 amino acids 6 to 166, obtained from GenBank: CAA51363.1) was cloned and expressed in the pPROEX HTa vector (Figure 1), and the resulting protein, rprM-DENV-2, weighing 21 kDa, was purified via affinity chromatography (Figure 2). The immunogenicity of this protein was assessed in a Wistar rat model following three doses of the recombinant protein. The presence of antigen-specific IgG antibodies capable of recognizing the native antigen was documented using ELISA (Figure 3) and confirmed after recognition of the virus in Vero cells previously infected with DENV-2 (Figure 4). With these results, the immunogenicity of the prM protein was verified, as reported by other authors in a Swiss mouse model, where they compared the response to a viral challenge [27], or in inoculated BALB/c mice, where they subsequently performed in vitro assays [28]. These studies support the use of the prM-DENV-2 protein as a tool in in vitro assays to evaluate DENV-2 infection models. They are consistent with the data obtained by Raktasha Munir et al., who documented the successful use of a prM recombinant protein from DENV-2 to evaluate anti-DENV-2 immune response for diagnostic and vaccination purposes; they reported the use of an antigen to evaluate IgG antibodies [23].

On the other hand, one advantage of using the prM protein compared with the E protein as the antigen in immunoassays is that prM is more conserved than the E protein among the four DENV serotypes (Supplementary Figure S2); therefore, it can be used more extensively than the E protein for screening purposes.

Additionally, we analyzed the usefulness of the recombinant protein rprM-DENV-2 in indirect ELISAs to detect antibody responses in samples from patients diagnosed with dengue (Figure 5). Paired saliva and serum samples were collected from the negative control group (residents of Mexico City) and from the group of patients diagnosed with dengue. Saliva sampling was considered a less invasive method for monitoring anti-DENV responses in patients for whom obtaining blood samples is difficult or who live in remote areas.

On the other hand, previous studies indicate that IgM, IgA, and IgG antibodies induced by primary or secondary DENV infections follow the same kinetics in both serum and saliva [14], and IgA antibodies in human serum are present at concentrations comparable to those of IgG3 and IgG2 [15,29,30]. In circulation, IgA is the second most abundant immunoglobulin class, and its concentration (approximately 2 mg/mL) is only behind that of IgG [31]. For these reasons, we decided to search for IgA antibodies in saliva and serum in this study.

Since these samples were obtained in patients with secondary infections, our results are consistent with those reported by Yap et al., who suggested that detecting IgA in saliva may represent a valuable diagnostic marker, particularly in secondary DENV infections [14]. This observation supports the use of the prM protein in vaccines and as a diagnostic method. However, further confirmation is required, as demonstrated by the studies conducted by Yap et al. and Andries et al., which documented the benefit of detecting IgA in saliva samples from DENV patients [14,32]. These observations could support the main finding of this work, the detection of specific IgA against prM in an immunoassay for monitoring purposes, which in this context means follow-up of clinical manifestations in patients or as a potential diagnostic tool.

Subsequently, we compared paired saliva and serum samples and found a significant difference (*p* < 0.0001), with higher EU/mL values in serum than in saliva. This finding is consistent with reports indicating that IgA is the most heterogeneous immunoglobulin isotype, and this variation is related to the fact that IgA is not only found in the circulation and tissues but is also the predominant immunoglobulin in external secretions, in the form of secretory IgA (S-IgA) [31]. Secretory IgA exerts distinct biological activities from IgA present in circulation and tissues. In adult serum, IgA is found in concentrations ranging from 0.7 to 3.4 mg/mL [33] and is present mainly (>80%) as monomeric IgA (mIgA). The remaining fraction of serum IgA is present in polymeric form, mainly dimeric [31].

However, when we analyzed IgA against the rprM-DENV-2 antigen, dividing samples from patients diagnosed with dengue into two subgroups (No WS and WS), we found no significant difference in either serum or saliva. This contrasts with the results for other antigens; for example, IgA antibodies against the E protein counteract the ADE phenomenon, while IgA antibodies against the non-structural protein 1 (NS1) are present in severe disease. It has even been shown that IgA antibodies against the NS1 protein can activate neutrophils in severe dengue infection [30]. In this study, we found that IgA antibodies against the prM protein are useful for diagnosing dengue in saliva and serum samples. A larger number of samples would need to be tested to corroborate our findings because so far, we have not been able to differentiate between infection without warning signs and infection with warning signs.

### Limitations of the Study

Although our data are consistent with studies that promote the use of rprM-DENV-2 as a diagnostic tool, more studies are needed to address some limitations identified in this study; for instance, it has been reported that non-glycosylated recombinant proteins produced in prokaryotic systems may alter conformational epitopes, which could go undetected by computational tools. This issue might influence the immunogenicity and antigenicity of viral immunogens [34]. Therefore, it is essential to compare the rprM-DENV-2 protein from both eukaryotic and prokaryotic sources, as well as to evaluate this protein alongside other viral proteins in the same context. Additionally, the results indicate that rprM-DENV-2 is effective for the non-invasive detection of salivary IgA antibodies. Due to the small clinical sample size, the study’s conclusions may be limited and should be interpreted as a proof-of-principle. A larger number of clinical samples per group is needed to validate these findings more effectively.

Furthermore, it has been reported that the prM protein induces strong cross-reactivity among the different serotypes of the DENV [8,35]. While this characteristic enhances the usefulness of the rprM-DENV-2 protein for the primary diagnosis of dengue infection, it may also pose a challenge for the specific identification of the DENV serotype. Additional evaluation is necessary to address this concern. Moreover, the specific DENV serotype that infected each patient was unknown. Finally, to obtain the entire landscape of the humoral response against dengue using the ELISA system that is proposed in this study, besides testing other viral proteins, approaches including the evaluation of other immunoglobulins; the classification of primary and secondary infections; and the collection of samples from different clinical presentations (asymptomatic, dengue with No WS, dengue with WS, and severe dengue) would be most appropriate to address the complexity of this infectious disease.

Other studies have evaluated IgA against dengue viral antigens such as protein E and NS1, highlighting the relevance of this antibody in the context of the infection [30].

Finally, to the best of our knowledge, our study is the first to evaluate the DENV-IgA response using prM as an antigen in an immunoassay and to explore its impact on the immune response to dengue for potential monitoring or diagnostic purposes.

## 4. Materials and Methods

### 4.1. Cells and Virus

Mosquito C6/36 cells (ATCC CRL-1660 (Manassas, VA, USA)) derived from *Aedes albopictus* were grown in MEM (Sigma-Aldrich, Burlington, MA, USA) supplemented with 10% fetal bovine serum (FBS) (Biowest, Nuaillé, Pays De La Loire, France) at 34 °C; Vero cells (ATCC CRL-1586 (Manassas, VA, USA)), a kidney epithelial cell line derived from an African Green Monkey, were grown in Roswell Park Memorial Institute (RPMI) media, pH 7.8 (Biowest, Nuaillé, Pays De La Loire, France), supplemented with 5% FBS and 1% of antibiotic-antimycotic solution (Biowest, Nuaillé, Pays De La Loire, France) at 37 °C. The virus used in this study belongs to serotype 2 (DENV-2) and shares high homology with the New Guinea strain (GenBank AF038403.1). It was obtained in 1997 from a Mexican patient from the East Coast and was provided by the Instituto de Diagnóstico y Referencia Epidemiológicos (InDRE) from Mexico.

### 4.2. Propagation and Purification of DENV-2

The viral stock was prepared by infecting a monolayer of C6/36 cells in 75 cm^2^ cell culture flasks at 75% to 85% confluence. The flasks were monitored daily via microscopy; once a cytopathic effect was observed, the contents were homogenized and diluted with a solution of 40% polyethylene glycol in 2 M NaCl (Sigma-Aldrich, St. Louis, MO, USA), and the homogenate was incubated overnight at 4 °C. Subsequently, the homogenate was centrifuged at 6000 rpm for 1 h at 4 °C; the supernatant was removed, and the pellet was resuspended in a glycine buffer solution (50 mM Tris, 200 mM glycine, 100 mM NaCl, and 1 mM EDTA (Sigma-Aldrich, St. Louis, MO, USA)) at a 1:15 ratio (*v*/*v*), then supplemented with FBS at a 1:30 ratio (*v*/*v*). The virus-containing solution was homogenized again, aliquoted into various volumes, and stored at −70 °C.

### 4.3. Gene Synthesis and Plasmid Construction

The amino acid sequence for the DENV prM protein spanned aa residues 6 to 166 from GenBank: CAA51363.1. The DENV-2 viral genome was obtained from infected C6/36 cells (showing 70–80% cytopathic effect) treated with TRIZOL reagent (GIBCO, Grand Island, NY, USA); the suspension was homogenized and centrifuged at 12,000× *g* for 15 min at 4 °C, after which the aqueous phase containing the RNA was separated. The RNA served as a template for reverse transcription using the Superscript III reverse transcriptase kit (Invitrogen, Carlsbad, CA, USA) to generate DENV cDNA. The sequence encoding the DENV-2 prM protein was amplified from the cDNA using the following primers: CCG GAA TTC TTC CAT TTA ACC ACA CGT (5′) and CCT TCA ATG ACA TTG CGG CCG CTT (3′); the resulting PCR product and the pProEx Hta plasmid were digested with EcoRI and NotI enzymes and purified. Then, the PCR product was cloned into the pProEx Hta plasmid downstream of the inducible *trc* promoter, flanked by EcoRI and NotI sites. The constructed plasmid was designated pProEx/prM/DENV-2 and purified from *Escherichia* *coli* (*E. coli* DH5α) using the EndoFree Plasmid Kit (Qiagen Inc., Valencia, CA, USA). To confirm that the desired sequence had been obtained, the construct was sequenced.

### 4.4. Expression of rprM-DENV-2 Protein

To analyze the expression of rprM-DENV-2, *E. coli* BL21 STAR (DE3) was transformed with plasmid pProEx/prM/DENV-2 and then cultured in Luria–Bertani medium (Difco Laboratories, Detroit, MI, USA) at 37 °C with 100 μg/mL ampicillin. A colony was isolated and grown under the previously mentioned conditions with agitation at 200 rpm until an optical density of 0.6 at 600 nm was reached; the inducer for the *trc* promoter, isopropyl-β-D-1-thiogalactopyranoside (IPTG; Sigma-Aldrich, Burlington, MA, USA), was added at 1 mM, and the culture was incubated again. The induction kinetics of prM protein expression were analyzed by taking aliquots at 0, 3, 6, and 12 h post-induction, alongside a non-induced control. Finally, all aliquots were adjusted to an OD at 600 nm of 0.6.

#### 4.4.1. Identification of rprM-DENV-2 Protein

From the adjusted aliquots of the induced bacteria, a 1 mL sample was taken from each and centrifuged at 13,000 rpm for 1 min; subsequently, the supernatant was removed, and the pellet was resuspended in 50 µL of 6X Laemmli loading buffer (Tris-HCl pH 6.8, SDS, glycerol, bromophenol blue, and β-mercaptoethanol), vortexed, and boiled for 10 min. These samples were run on a 12% SDS-PAGE gel, and stained with Coomassie blue. To confirm protein identity at different stages of production, 30 μL of each sample was resolved on an SDS-PAGE gel and then electrotransferred (120 V for 1.5 h) onto a nitrocellulose membrane (Hybond ECL; GE Healthcare, Chicago, IL, USA). The membrane was blocked with non-fat skim milk and then incubated with a primary antibody (mouse anti-His) (Thermo Fisher Scientific, Waltham, MA, USA), followed by a horseradish peroxidase (HRP)-conjugated secondary antibody (anti-mouse) (Thermo Fisher Scientific). After further washing three times with 0.05% PBS-Tween20 (PBST) and once with PBS, the immune reaction was visualized using a Pierce^®^ Western Lightning-enhanced chemiluminescence reagent (Thermo Fisher Scientific, Waltham, MA, USA), unless diaminobenzidine had been declared as the chromogen. The signal was detected by Chemi-Doc equipment (BioRad, Hercules, CA, USA).

#### 4.4.2. Purification of rprM-DENV-2 Protein

The bacterial culture was incubated with the inducer for 12 h; after this time, it was centrifuged at 6000 rpm for 20 min, the supernatant was removed, and the pellet was frozen for 2 h. Subsequently, 40 mL of lysis solution (100 mM NaH_2_PO_4_, 10 mM Tris-HCl, 8 M urea, pH 6.3) was added to the pellet; the mixture was agitated for 1 h and then centrifuged at 14,000 rpm for 30 min at 25 °C. The supernatant was collected, supplemented with 100 mM phenylmethylsulfonyl fluoride (as a protease inhibitor), and set aside. Separately, 500 μL of Ni-NTA agarose beads (Talon^®^, Clontech, San Jose, CA, USA) was washed three times with the lysis solution. Next, the agarose beads were mixed with the previously collected supernatant. An empty nickel column was loaded with this mixture and kept under gentle agitation for 1 h at 4 °C to allow the imidazole groups of the 6x-His tag to interact with the nickel ions on the agarose beads. After this time, the column was fixed in place, and the lower lid was removed to allow flow-through and the removal of unbound proteins. The rprM-DENV-2 protein (bound to the agarose beads) was eluted with 2 mL of elution solution (500 mM NaH_2_PO_4_, 50 mM NaCl, 50 mM imidazole, pH 8.0), and aliquots were collected. Finally, the eluates were analyzed by SDS-PAGE with Coomassie blue staining, Western blot, and dot blot.

#### 4.4.3. Conformation of rprM-DENV-2 Protein

To test that rprM-DENV-2 had a similar conformation to the native viral protein, another identification technique was performed: a dot blot on a PVDF membrane using samples from the previous purification step of rprM-DENV-2. The antibody used was a commercial mouse anti-prM-DENV monoclonal antibody (Abcam, Boston, MA, USA) that recognizes a conformational epitope of the prM protein, and as a detection antibody, an anti-mouse IgG1-HRP (Boehringer Ingelheim, Ingelheim am Rhein, Rheinland-Pfalz, Germany) was used. The membrane was treated with luminol, and then a radiographic film was exposed to the membrane, and Gel-Doc equipment (BioRad) was used to record this.

### 4.5. Indirect Evaluation of Antigenic Properties of the Protein rprM-DENV-2

#### 4.5.1. Production of Hyperimmune Rat Serum

Previously, to perform an immunization assay, an aliquot of 12.5 µL of the protein was diluted with 37.5 µL of sterile PBS to obtain 100 µg of rprM-DENV-2 protein per dose, then mixed with 50 µL of Freund’s complete adjuvant. Three female Wistar rats weighing 250 g were inoculated intraperitoneally with 100 µL of the formulated protein every two weeks, for a total of three doses.

Serum samples were then analyzed by enzyme-linked immunosorbent assay (ELISA). Briefly, the plate was covered with 5 µg/mL of rprM-DENV-2 and blocked with 5% non-fat skim milk in PBST (*w*/*v*), and serum samples from immunized rats at two-fold dilutions ranging from 1:50 to 1:400 were added and incubated for 1 h, at room temperature. Horseradish peroxidase (HRP)-conjugated rabbit anti-rat IgG was used as the secondary antibody. Hydrogen peroxide was used as the enzyme substrate, and *ortho*-phenylenediamine as chromogen, and the reaction was quenched with 2N H_2_SO_4_. The plate was read at 490 nm in a MultiScan reader (Thermo Scientific, Waltham, MA, USA).

#### 4.5.2. In Vitro Detection of Viral Infection Using the Anti-rprM-DENV-2 Sera

Vero cells were seeded on glass coverslips (6 × 10^4^) (Bellco, Vineland, NJ, USA) in a 24-well plate. Monolayers were infected with DENV-2 or incubated with a UV-inactivated virus (mock) with an MOI of 2. Then, cells were incubated at 37 °C for 18 h and treated with 10 μg/mL of brefeldin (BFA) (Sigma-Aldrich) to stop the vesicular traffic. At 24 h post-infection; the cells were fixed with 4% *para*-formaldehyde (Sigma-Aldrich, St. Louis, MO, USA) in PBS for 20 min at room temperature, then permeabilized with a solution of 0.1% Triton-X100 in PBS and blocked with 10% normal goat serum. The cell monolayer was incubated for 60 min with primary serum samples (1:100 dilution) from the immunized rats; after washing, Alexa Fluor 488-conjugated goat anti-rat IgG was used (Thermo Fisher Scientific). Finally, the nuclei were labeled with DAPI (1 μg/mL) in PBS for 10 min, and the slides were mounted with Vecta-shield plus DAPI (Vector Laboratories, Newark, CA, USA). The samples were examined under a fluorescence microscope (Leyca, SP2 model, Barcelona, Spain), and 40× photographs were taken.

### 4.6. rprM-DENV-2 Protein Antigenicity and Its Usefulness as an Antigen in an ELISA

#### 4.6.1. Human Samples

Saliva sample collection. Unstimulated saliva samples were obtained using the passive drool technique. Before collection, participants fasted from food, drinks, tobacco, and gum for at least 30 min. Then, around 500 µL of saliva was collected in sterile polypropylene tubes. The samples were transported to the lab under refrigeration (2–8 °C), centrifuged at 3000× *g* for 15 min at 4 °C to remove cell debris, then frozen at −80 °C for 2 h, thawed, and immediately centrifuged at 10,000× *g* for 15 min at 4 °C to remove mucins. The supernatant was recovered in sterile tubes and stored until analysis.

Twenty paired serum and saliva samples from patients with a clinical diagnosis of dengue, obtained from a previous study, were analyzed. Briefly, the samples were collected at the Regional High-Specialty Hospital of Veracruz during an outbreak occurring between June and August 2014. All DENV serotypes circulated in Veracruz during the study period, with serotype 2 being the most frequent [36,37]. Screening and confirmatory serological test were carried out with a SD BIOLINE Dengue Duo rapid kit (NS1 Ag/IgM/IgG) (Standard Diagnostic Inc., Kyonggi-do, Republic of Korea) and Panbio Dengue IgM and IgG capture (PanBio Diagnostics, Brisbane, Australia), respectively [38]. The samples were divided into two groups based on the presence or absence of warning signs in the patients, resulting in ten samples per group: Group 1 (samples from patients without warning signs, No WS) and Group 2 (samples from patients with warning signs, WS) (See File S1. Raw data file).

Eighteen paired serum and saliva samples from Mexico City residents with no history of clinical signs suggestive of dengue or other infectious diseases (and no febrile symptoms) in the preceding six months were analyzed as negative controls.

#### 4.6.2. Antigen Titration

As a control antigen, another structural protein, the recombinant envelope (rE) from all DENV serotypes, was used (PanBio Diagnostics, Brisbane, Australia), and the test antigen was the purified rprM-DEN-2 obtained in this study. First, the titration of the rE was carried out using serial double dilutions ranging from 1:25 to 1:1600 of the commercial antigen mixture; the dilutions were prepared in 0.1 M carbonate buffer, pH 9.6, and used to coat a 96-well plate overnight at 4 °C. Blocking was performed with 0.5% bovine serum albumin in PBST. Plates were washed five times with PBST between each step. Human serum from a DENV-positive case was used as the primary antibody, and anti-human IgG-HRP was used as the secondary antibody. A dilution of 1:200 was enough to saturate the system; therefore, this dilution was used for the rest of the experiments. While rprM-DENV-2 titration was carried out almost identically, using the system’s saturation as the criterion for selecting the concentration, the antigen concentration used to sensitize the plate was 5 μg/mL.

#### 4.6.3. IgA Determination

Once the systems were standardized, the human samples (serum or saliva) were diluted as needed in the blocking solution. HRP-labeled goat anti-human IgA (KPL, Gaithersburg, MD, USA) was used as a secondary antibody. Bound antibodies were detected using tetramethylbenzidine at room temperature in the dark for 15 min, with 0.1 M HCl as the stop solution. OD at 450 nm was measured with a microplate reader (Awareness Technology Inc., Palm City, FL, USA).

Endpoint titers were calculated using linear regression equations of the reciprocals of the sample dilutions that yielded OD values at 450 nm of 0.2 above the blank. Titers were expressed in EU/mL.

### 4.7. Statistical Analysis

Data were analyzed using GraphPad Prism version 7 software (San Diego, CA, USA). A multiple *t*-test was performed to assess the kinetics of anti-rprM-DENV-2 antibody induction in rats. Results of IgA antibody detection in human samples are expressed as EU/mL. Data normality was assessed using the Shapiro–Wilk test. Independently collected data with a non-normal distribution were analyzed using the Mann–Whitney test. For paired data with a non-normal distribution, the Wilcoxon matched-pairs signed-rank test was used. A *p*-value <0.05 was considered statistically significant.

ROC curves [39] were obtained using the software GraphPad V11.0.2.92 (San Diego, CA, USA). The AUROC was determined using a 95% confidence interval. An AUROC >0.7 was considered acceptable. The cut-off, *p*-value, sensitivity, and specificity were calculated based on Youden index method [39].

The PPV and NPV were calculated as described previously [38].

## 5. Conclusions

Our findings confirm that the rprM-DENV-2 protein retains the antigenicity of native protein, since it induces IgG antibodies that could be used in the in vitro analysis of cellular infection. Additionally, we can discriminate IgA antibodies against the rprM-DENV-2 protein in saliva and serum samples from patients with DENV infection from those of negative donor samples. We highlight that saliva is a bodily fluid that can be obtained non-invasively and could be useful in a potential screening test for detecting all DENV serotypes.

## Figures and Tables

**Table 1:** Analysis of ELISA (sensitized with the rprMDENV-2 antigen) results in saliva samples from patients diagnosed with dengue.

Data Analyzed	Patients	Negative Controls	Total
Positives	16	0	
Negatives	4	18	22
Total	20	18	38

Cut-off: 103.6 EU/mL, PPV = 100%, NPV = 81.8%. See Section 4.7.

**Table 2:** Analysis of ELISA (sensitized with the rprMDENV-2 antigen) results in serum samples from patients diagnosed with dengue.

Data Analyzed	Patients	Negative Controls	Total
Positives	20	0	20
Negatives	0	18	18
Total	20	18	38

Cut-off: 198.1 EU/mL, PPV = 100%, NPV = 100%. See Section 4.7.

## Data Availability

The data presented in this study are available in the body of this paper.

## Supplementary Materials

The following supporting information can be downloaded at: https://www.mdpi.com/article/10.3390/ijms27188087/s1.

## Abbreviations

The following abbreviations are used in this manuscript:

ADE	Antibody-dependent enhancement
ATCC	American Type Culture Collection
AUROC	Area under the ROC
BFA	Brefeldin
BSA	Bovine serum albumin
cDNA	Complementary deoxyribonucleic acid
DNA	Deoxyribonucleic acid
DENV	Dengue virus
E	Envelope protein
*E. coli*	*Escherichia coli*
ELISA	Enzyme-linked immunosorbent assay
EU/mL	ELISA units per milliliter
FBS	Fetal bovine serum
HRP	Horseradish peroxidase
IgA	Immunoglobulin A
IgG	Immunoglobulin G
IgM	Immunoglobulin M
IPTG	Isopropyl β-D-1-thiogalactopyranoside
MOI	Multiplicity of infection
NPV	Negative Predictive Value
NS1	Non-structural protein 1
No WS	Without warning signs
OD	Optical density
PCR	Polymerase Chain Reaction
PPV	Positive Predictive Value
prM	Premembrane protein
RPMI	Roswell Park Memorial Institute
ROC	Receiver Operating Characteristic
SDS-PAGE	Sodium Dodecyl Sulfate Polyacrilamide Gel Electrophoresis
WHO	World Health Organization
WS	With warning signs
