# Supplementary material for: Expression and Immunogenic Application of Recombinant DENV-2 rprM Protein for the Non-Invasive Detection of Salivary IgA Antibodies
Source: Int J Mol Sci. 2026 Sep 11;27(18):8087. doi: 10.3390/ijms27188087 (PMC13606918; doi:10.3390/ijms27188087)
Supplement: Supplementary file 1 [file ijms-27-08087-s001.zip › Suplementary Figure S1.pdf]

A)

```
(Threshold=0.5)
----------------------------------------------------------------------
SeqName      Position  Potential   Jury    N-Glyc
                               agreement  result
----------------------------------------------------------------------
Sequence        7 NGEP    0.2339     (9/9)    ---
Sequence       32 NMCT    0.5680     (8/9)    +
Sequence       59 NEPE    0.7154     (9/9)    ++
Sequence       69 NFTS    0.5860     (8/9)    +      SEQUON ASN-XAA-SER/THR.
----------------------------------------------------------------------
```

B)

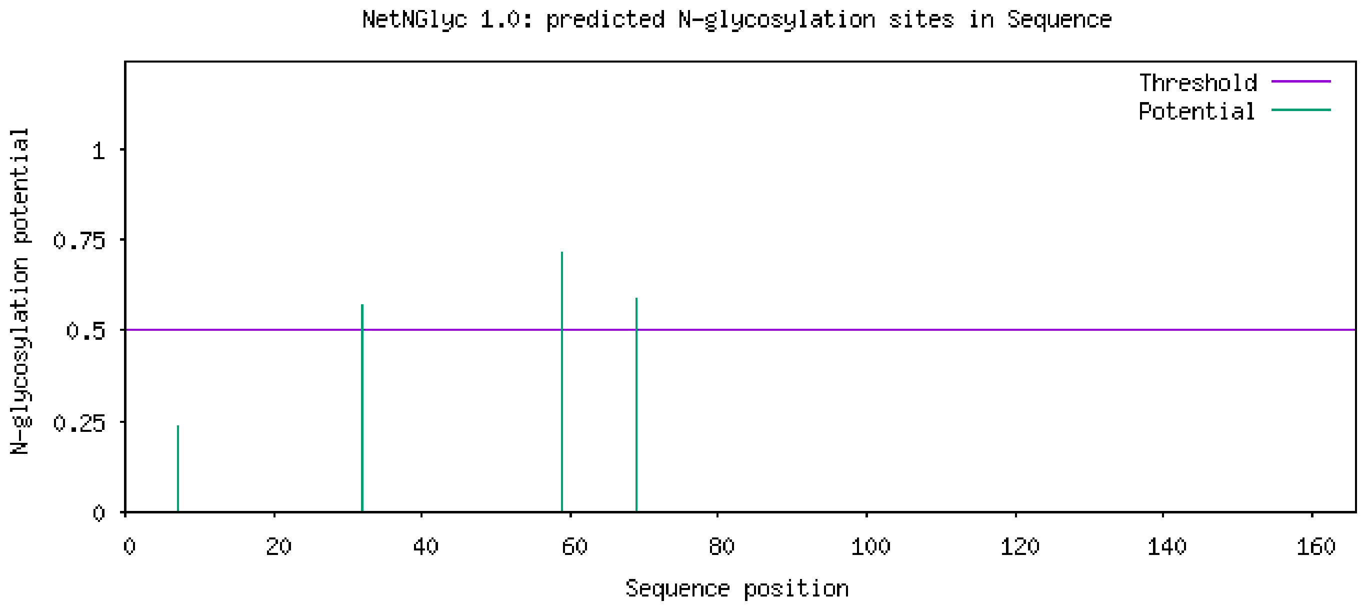

Graphics in PostScript

Supplementary Figure S1. Evaluation of the glycosylation degree of prM protein. The sequence of prM DENV-2 was submitted to the NetNglyc server to predict N Glycosylation sites using artificial neural networks that examine the sequence context of Asn-Xaa-Ser/Thr sequons (A). The result is graphed (B).
