# Supplementary material for: Expression and Immunogenic Application of Recombinant DENV-2 rprM Protein for the Non-Invasive Detection of Salivary IgA Antibodies
Source: Int J Mol Sci. 2026 Sep 11;27(18):8087. doi: 10.3390/ijms27188087 (PMC13606918; doi:10.3390/ijms27188087)
Supplement: Supplementary file 1 [file ijms-27-08087-s001.zip › Suplementary Figure S2.pdf]

A)

```
prMDENV-4      fhlstrdgeplmivakhergrpllfkttteginkctliamdlgemcedtvtykcpllvnte     60
prMDENV-3      fhltsrdgeprmivgknergksllfktasginmctliamdlgemcddtvtykcpliaeve     60
prMDENV-1      fhlttrggephmivskqergksllfktsagvnmctliamdlgelcedtmtykcpriteae     60
prMDENV-2      FHLTTRNGEPHMIVSRQEKGKSLLFKTEDGVNMCTLMAMDLGELCEDTITYNCPLLRQNE     60
               ***::*.*** ***.::*:*: *****  *:* ***:******:*:**:**:** : : *

prMDENV-4      pedidcwcnltstwvmygtctqsgerrrekr  91
prMDENV-3      pedidcwcnltstwvtygtcnqagehrrdkr  91
prMDENV-1      pddvdcwcnatdtwvtygtcsqtgehrrdkr  91
prMDENV-2      PEDIDCWCNSTSTWVTYGTCTATGEHRREKR  91
               *:*:***** *.*** ****. :**:**:**
```

B)

Phylogenetic Tree

This is a Neighbour-joining tree without distance corrections.

Branch length: ◉ Cladogram ○ Real

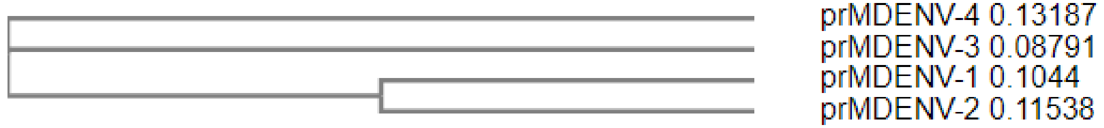

Supplementary Figure S2. Homology of the prM protein among four Dengue serotypes. Aminoacid sequences of prM protein from 4 serotypes were submitted to MAFT (Multiple Alignment using Fast Fourier Transform) Clustal W. *indicate homology (A). Phylogenetic analysis of prM protein from 4 serotypes (B).
